# Could Dampening Expression of the Neisseria gonorrhoeae *mtrCDE*-Encoded Efflux Pump Be a Strategy To Preserve Currently or Resurrect Formerly Used Antibiotics To Treat Gonorrhea?

**DOI:** 10.1128/mBio.01576-19

**Published:** 2019-08-13

**Authors:** Shaochun Chen, Kristie L. Connolly, Corinne Rouquette-Loughlin, Alexander D’Andrea, Ann E. Jerse, William M. Shafer

**Affiliations:** a Department of Microbiology and Immunology and the Emory Antibiotic Resistance Center, Emory University School of Medicine, Atlanta, Georgia, USA; b Laboratories of Bacterial Pathogenesis, VA Medical Center, Decatur, Georgia, USA; c National Center for STD Control, Chinese Center for Disease Control and Prevention, Nanjing, China; d Institute of Dermatology, Chinese Academy of Medical Sciences and Peking Union Medical College, Nanjing, China; e Department of Microbiology and Immunology, F. Edward Hebert School of Medicine, Uniformed Services University, Bethesda, Maryland, USA; Duke University School of Medicine; University of Oklahoma; University of Iowa

**Keywords:** *Neisseria gonorrhoeae*, efflux pumps, penicillin, transcriptional regulation

## Abstract

Neisseria gonorrhoeae has developed resistance to every antibiotic introduced for treatment of gonorrhea since 1938, and concern now exists that gonorrheal infections may become refractory to all available antibiotics approved for therapy. The current recommended dual antibiotic treatment regimen of ceftriaxone (CRO) and azithromycin (AZM) is threatened with the emergence of gonococcal strains displaying resistance to one or both of these antibiotics. Non-beta-lactamase resistance to penicillin and third-generation cephalosporins, as well as low-level AZM resistance expressed by gonococci, requires overexpression of the *mtrCDE*-encoded efflux pump, which in wild-type (WT) strains is subject to transcriptional repression by MtrR. Since earlier studies showed that loss of MtrCDE renders gonococci hypersusceptible to beta-lactams and macrolides, we hypothesized that transcriptional dampening of *mtrCDE* would render an otherwise resistant strain susceptible to these antibiotics as assessed by antibiotic susceptibility testing and during experimental infection. In order to test this hypothesis, we ectopically expressed a WT copy of the *mtrR* gene, which encodes the repressor of the *mtrCDE* efflux pump operon, in N. gonorrhoeae strain H041, the first reported gonococcal strain to cause a third-generation-cephalosporin-resistant infection. We now report that MtrR production can repress the expression of *mtrCDE*, increase antimicrobial susceptibility *in vitro*, and enhance beta-lactam efficacy in eliminating gonococci as assessed in a female mouse model of lower genital tract infection. We propose that strategies that target the MtrCDE efflux pump should be considered to counteract the increasing problem of antibiotic-resistant gonococci.

## Abstract (precis)

The emergence of gonococcal strains resistant to past or currently used antibiotics is a global public health concern, given the estimated 78 million infections that occur annually. The dearth of new antibiotics to treat gonorrhea demands that alternative curative strategies be considered to counteract antibiotic resistance expressed by gonococci. Herein, we show that decreased expression of a drug efflux pump that participates in gonococcal resistance to antibiotics can increase gonococcal susceptibility to beta-lactams and macrolides under laboratory conditions, as well as improve antibiotic-mediated clearance of gonococci from the genital tract of experimentally infected female mice.

## INTRODUCTION

Gonorrhea remains a major global public health concern, given the estimated 78 million infections that occurred in 2012 (1). Furthermore, the emergence of Neisseria gonorrhoeae clinical isolates displaying clinically significant levels of resistance to currently or previously used antibiotics and the lack of new antibiotics entering clinical practice in the foreseeable future has raised the concern that some infections may soon be untreatable (2, 3). With the lack of a vaccine, society relies on antibiotics to not only cure a gonorrheal infection but also reduce spread of the gonococcus in the community (4).

Research on the mechanisms by which bacteria, including the gonococcus, develop resistance to antibiotics can provide insights as to heretofore-unexploited targets for drug discovery. In this respect, the realization that overexpression of multidrug efflux pumps can contribute to bacterial resistance to antibiotics prompted early attempts to develop efflux pump inhibitors (EPIs). Unfortunately, initial efforts that identified promising compounds were abandoned over a decade ago due to host cell toxicity concerns (reviewed in reference 5). However, with the worldwide public health concern about antibiotic resistance and the dearth of new antibacterials, especially those that recognize unique targets or metabolic pathways, a renewed effort to develop EPIs should be considered.

**FIG 1:**
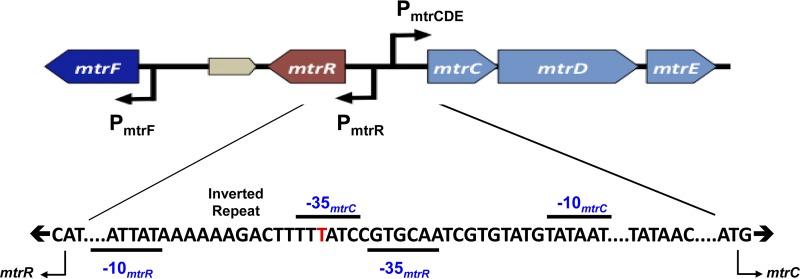
Shown is the organization of the *mtr* locus in N. gonorrhoeae strain FA19. Promoters are indicated by a bent arrow in the direction of transcription. The intergenic region between *mtrR* and *mtrCDE* is expanded to show the overlapping *mtrR* and *mtrCDE* promoters, with the 13-bp inverted repeat (5′-AAAAAGACTTTTT-3′) positioned between the −10 and −35 *mtrR* promoter hexamers indicated. The red T highlights where a single base pair deletion occurs in strain H041 and other gonococci that overexpress *mtrCDE* (7). The deletion results in abolishment of *mtrR* expression and significantly elevates gonococcal resistance to certain antimicrobials that are substrates of the MtrCDE efflux pump (11).

Previous work has shown that the resistance-nodulation-division (RND) efflux pump termed MtrCDE (Fig. 1) is needed for clinically defined levels of gonococcal resistance to certain antibiotics, including beta-lactams and macrolides (6–10). Overexpression of the *mtrCDE* operon in clinical isolates or laboratory-derived genetic variants due to *cis*-acting promoter mutations can significantly decrease gonococcal susceptibility to antimicrobials (9, 11, 12). Importantly, Golparian et al. (7) showed that loss of MtrCDE in such overexpressing strains can render gonococci clinically sensitive to penicillin (PEN), as defined by the MIC breakpoint. In addition to PEN, loss of MtrCDE in clinical isolate H041, the first strain to cause an extended-spectrum-cephalosporin (ESC)-resistant case of gonorrhea (13), increased gonococcal susceptibility to cefixime (CFM), ceftriaxone (CRO), and azithromycin (AZM) 4- to 8-fold (7).

10.1128/mBio.01576-19.1 FIG S1 Dose response for *in vivo* clearance of S6. Download FIG S1, PDF file, 0.6 MB. Copyright © 2019 Chen et al. 2019 Chen et al. This content is distributed under the terms of the Creative Commons Attribution 4.0 International license.

In addition to *cis*-acting promoter mutations, loss of the MtrR repressor, which negatively controls *mtrCDE* expression (14), can increase gonococcal resistance to antimicrobials, including classical antibiotics and host-derived compounds that participate in innate defense against infection (reviewed in reference 6). In strains having a single base pair deletion in the *mtrR* promoter (e.g., strain H041) (Fig. 1), the most common *cis*-acting mutation, the expression of *mtrR* is abrogated (11). Thus, we hypothesized that ectopic expression of *mtrR* in these strains would decrease levels of MtrCDE and render gonococci more susceptible to antibiotics both under laboratory conditions and during experimental infection. The results reported herein suggest that strategies that dampen efflux pump gene expression could allow for a return of previously used antibiotics (e.g., PEN) or help preserve those (CRO) currently used to treat infections like gonorrhea.

## RESULTS AND DISCUSSION

### Ectopic expression of *mtrR* in strain H041 decreases gonococcal resistance to antibiotics recognized by the MtrCDE efflux pump.

Strain H041 caused the first reported case of ESC-resistant gonorrhea and has been extensively studied regarding antibiotic resistance determinants (13). With respect to determinants causing beta-lactam resistance, H041 possesses a mosaic-like *penA* gene that encodes an extensively remodeled penicillin-binding protein 2 (PBP2) that is poorly acylated by beta-lactams, point mutations in *ponA* and *porB* that reduce the affinity of PBP1 for beta-lactams and the influx of antibiotics, respectively, and a single base pair deletion in the promoter that drives transcription of *mtrR* (reviewed in reference 15). Previous work with other gonococcal strains showed that this base pair deletion also abrogates the expression of *mtrR* while elevating the expression of the *mtrCDE* operon (11). Importantly, loss of the MtrCDE efflux pump in H041 by gene inactivation resulted in increased susceptibility of the strain to beta-lactams and macrolides (7). Thus, we hypothesized that ectopic expression of *mtrR* would result in enhanced susceptibility of this strain to beta-lactams and macrolides.

**FIG 2:**
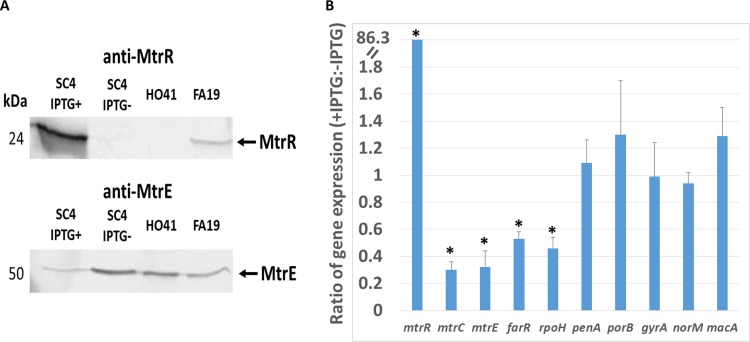
Inducible, ectopic expression of WT *mtrR* represses expression of *mtrCDE* and other MtrR-regulated genes. (A) Western blot analyses of whole-cell lysates of strains FA19, H041, and SC4 (with or without IPTG) probed for MtrE and MtrR. (B) Summary of fold changes of expression levels of representative antibiotic resistance genes in SC4 as the ratios of qRT-PCR results from RNA extracted from IPTG-treated versus -untreated cultures; specific values and statistical significance values are provided in Table S1. Asterisks indicate statistically significant results.

To test this hypothesis, we inserted a wild-type (WT) copy of the *mtrR* gene, which encodes an active MtrR repressor protein that dampens the expression of *mtrCDE* (16), from the antibiotic-sensitive strain FA19 into the *lctP-aspC* chromosomal region of strain H041 using pGCC4 (see Materials and Methods); the expression of cloned genes in pGCC4 is directed by an isopropyl-β-d-thiogalactopyranoside (IPTG)-inducible *lac* promoter (17). A resulting transformant (strain SC4) was used to determine the impact of MtrR expression on beta-lactam resistance. We found that growth of SC4 in the presence of IPTG resulted in increased levels of MtrR and decreased amounts of the MtrE outer membrane protein channel of the MtrCDE efflux pump (Fig. 2A). This result was verified by transcriptional profiling studies that used quantitative real-time reverse transcription-PCR (qRT-PCR) to quantify transcript levels of *mtrR*, *mtrC*, and other MtrR-repressed genes (*farR* and *rpoH*) in SC4 grown in the absence or presence of IPTG. Briefly, this analysis showed that growth of SC4 in the presence of IPTG resulted in a nearly 80-fold increase in *mtrR* expression and 3- to 5-fold decreases in all MtrR-repressed genes tested, but not in genes outside the MtrR regulon (Fig. 2B and Table S1 in the supplemental material) (18).

10.1128/mBio.01576-19.2 TABLE S1 Gene expression ratio of different genes under the induction of IPTG/without IPTG in SC4. Download Table S1, PDF file, 0.3 MB. Copyright © 2019 Chen et al. 2019 Chen et al. This content is distributed under the terms of the Creative Commons Attribution 4.0 International license.

Consistent with changes in gonococcal gene expression due to ectopic overexpression of *mtrR*, we found that susceptibility of SC4 to a known MtrCDE efflux pump substrate, Triton X-100 (TX-100) (11), was greatly increased under inducible conditions (Table 1). To verify that the MtrCDE efflux pump was involved in the IPTG-inducible TX-100 susceptibility of strain SC4, we constructed a transformant of SC4 (strain SC5) that contained an insertionally inactivated *mtrD* gene. We found that strain SC5 was 500-fold more susceptible to TX-100 than the parental strain SC4 regardless of the presence of IPTG (Table 1). Based on this result and past studies showing the importance of the MtrCDE efflux pump in participating in beta-lactam resistance (7, 8), we assessed whether inducible expression of *mtrR* would increase susceptibility to penicillin and ceftriaxone. As shown by the results in Table 1, incubation of SC4 in the presence of IPTG resulted in a 2- and an 8-fold increase in susceptibility to CRO and PEN, respectively. As a control, we also constructed a derivative of H041 that expressed a mutated version of MtrR (MtrR G45D, bearing a change of G to D at position 45) from the *lac* promoter in pGCC4. Previous studies showed that MtrR G45D (encoded by *mtrR45* allele) lacked DNA-binding activity and that its presence relieved repression of *mtrCDE*, resulting in increased antimicrobial resistance compared to that of the otherwise WT parent (9, 14). However, unlike ectopic expression of WT *mtrR*, similar expression of *mtrR45* in H041 in the presence of IPTG did not decrease resistance to PEN and TX-100 (data not presented). Thus, we conclude that dampening the expression of *mtrCDE* by expressing WT MtrR in an ESC-resistant gonococcal strain can result in enhanced bacterial susceptibility to beta-lactam and macrolide antibiotics.

**TABLE 1:** Gonococcal strains and antimicrobial susceptibilities

Strain	MIC (μg/ml) without/with IPTG		
	CRO	PEN	TX-100
FA19	<0.008/<0.008	0.015/0.015	125/125
H041	2/2	8/8	>8,000/>8,000
SC4 (H041 *mtrR*^+^)	2/1	8/1	>8,000/62.5
SC5 (SC4 *mtrD*::*kan*)	1/1	0.5/0.5	16/16
SC6 (SC4 *rpsL*)	2/1	8/1	>8,000/62.5
SC7 (H041 *rpsL*)	2/2	8/8	>8,000/>8,000

### Ectopic expression of WT *mtrR* increases the ability of beta-lactams to clear infection caused by resistant gonococci.

To test whether ectopic expression of WT *mtrR* would increase the susceptibility of N. gonorrhoeae to CRO *in vivo*, we used modeled CRO pharmacokinetic (PK) data (19) to design treatment regimens that might be effective against beta-lactam-resistant gonococci. For this purpose, we used streptomycin (STR)-resistant (*rpsL*) versions of SC4 (SC6) and H041 (SC7) in a recently described treatment protocol using experimentally infected mice (19); the STR-resistant phenotype is necessary in the infection model, as STR is administered to mice prior to infection to help eliminate the normal bacterial flora (20). We recently showed that the estimated therapeutic time (the time that the free drug concentration remains above the MIC [*fT*_MIC_]) for CRO is ∼23 h against an ESC-susceptible strain (FA1090) and that PK data could be used to design a dosing strategy that would effectively clear H041 infection (19). No single dose of CRO was effective against this strain as predicted by the PK data. However, administration of a 120-mg/kg dose of CRO three times a day every 8 h (TID) cleared H041 infection in a majority (90%) of mice at 48 h posttreatment. This regimen was predicted to sustain plasma levels above the H041 MIC for 23.9 h.

**FIG 3:**
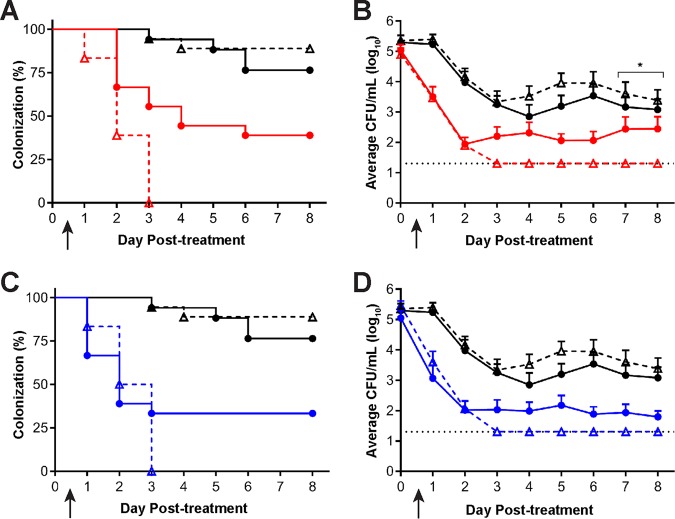
Administration of CRO eradicated vaginal infection in SC6-infected mice, but not in those infected with SC7. Mice infected vaginally with SC7 (circles) or SC6 (triangles) were treated on day 0 (arrow) with CRO using either a 60-mg/kg TID (red) or a 120-mg/kg BID (blue) dosing regimen (*n* = 17 or 18 mice/group). PBS TID (black) was administered as a negative control. (A) All mice infected with SC6 that received CRO in a 60-mg/kg TID dosing regimen cleared infection within 72 h posttreatment, and there was a significantly greater clearance rate in SC7-infected mice that received the 60-mg/kg TID regimen (*P = *0.0006). (B) There was a significant reduction in the average number of CFU/ml recovered from CRO-treated mice infected with either SC7 or SC6 compared to the average number recovered from mice in the PBS vehicle control group (*P < *0.01). There was a significant reduction in the average bacterial burden in SC6-infected mice that received CRO compared to the results for CRO treatment of SC7 mice on days 7 and 8 posttreatment (*, *P < *0.0001). (C) All SC6-infected mice that received 120 mg/kg CRO BID cleared infection within 72 h posttreatment, while in comparison, SC7-infected mice receiving this regimen remained 30% colonized through day 8 posttreatment. Groups infected with either strain that received CRO had a significant reduction in the number of infected mice compared to groups that received the corresponding PBS negative control (*P < *0.02). (D) There was a significant reduction in the average number of CFU/ml recovered from CRO-treated mice infected with either SC7 or SC6 when compared to the results for the PBS vehicle control group for each infection (*P < *0.0008).

Based on the CRO MICs of strains FA1090 and H041, we predicted that the CRO *fT*_MIC_ would need to be >22 h for SC6 and <22 h for SC7 to permit distinguishing the susceptibility of these two strains *in vivo*. The *fT*_MIC_ for 60 mg/kg CRO TID or 120 mg/kg CRO twice daily (BID) are predicted to be 14.7 and 18.1 h, respectively, for strain SC7, and we previously showed that these regimens were only moderately effective in clearing parent strain H041 infections (19). In contrast, the *fT*_MIC_ of both of these regimens for SC6 was predicted to be >24.9 h, and thus, they might be more effective against this strain. In two independent experiments, groups of BALB/c mice were experimentally infected with strain SC7 or SC6 as described in Materials and Methods. All mice had positive N. gonorrhoeae vaginal cultures on the 2 days after bacterial inoculation. Mice infected with each strain were given 120 mg/kg CRO BID, 60 mg/kg CRO TID, or phosphate-buffered saline (PBS) by intraperitoneal injection (*n* = 8 to 10 mice/group/experiment). Vaginal swab samples were quantitatively cultured for N. gonorrhoeae for 8 consecutive days starting 24 h after the first dose. Combined results from the two experiments are shown in Fig. 3. In both experiments, the percentages of SC6- or SC7-infected mice that cleared infection over 8 days posttreatment were significantly greater than the percentages in the corresponding PBS control groups for both treatment regimens (* P* ≤ 0.01) (Fig. 3A and C). All SC6-infected mice that were treated with either dosing regimen of CRO cleared the infection, while at least a third of SC7-infected mice remained infected for the remainder of the 8 days (Fig. 3A and C). Following the administration of 60 mg/kg CRO TID, 61% and 100% of SC6-infected mice cleared the infection within 48 and 72 h, respectively, compared to 33% and 44% of SC7-infected mice at these time points (SC6 versus SC7, *P* = 0.0006). However, there was no statistical difference in the efficacy of the 120-mg/kg CRO BID dose in clearing SC6 or SC7 infection (SC6 versus SC7, *P* = 0.4) (Fig. 3C), confirming our earlier work showing that CRO resistance can be overcome by delivering multiple, high doses of this antibiotic. We also analyzed the effects of different CRO dosing regimens on the gonococcal bioburden by comparing the numbers of CFU/ml recovered from vaginal swab sample suspensions before and after treatment. While both 60 mg/kg CRO TID and 120 mg/kg CRO BID significantly reduced the number of bacteria recovered posttreatment compared to the number in mice given PBS, only the mice treated with 60 mg/kg CRO showed a significant clearance of SC6 compared to the clearance of SC7 at later time points during infection (Fig. 3B), but there was no difference between mice infected with SC6 or SC7 when the 120-mg/kg CRO dose was used (Fig. 3D). A lower dose of CRO (30 mg/kg BID) was administered to determine if SC6 infection would be more susceptible than predicted by PK analysis, which gave a predicted *fT*_MIC_ of 14.7 h. This lower dose of CRO cleared SC6-infected mice as predicted by previous PK analysis and efficacy studies (Fig. S1) (19).

**FIG 4:**
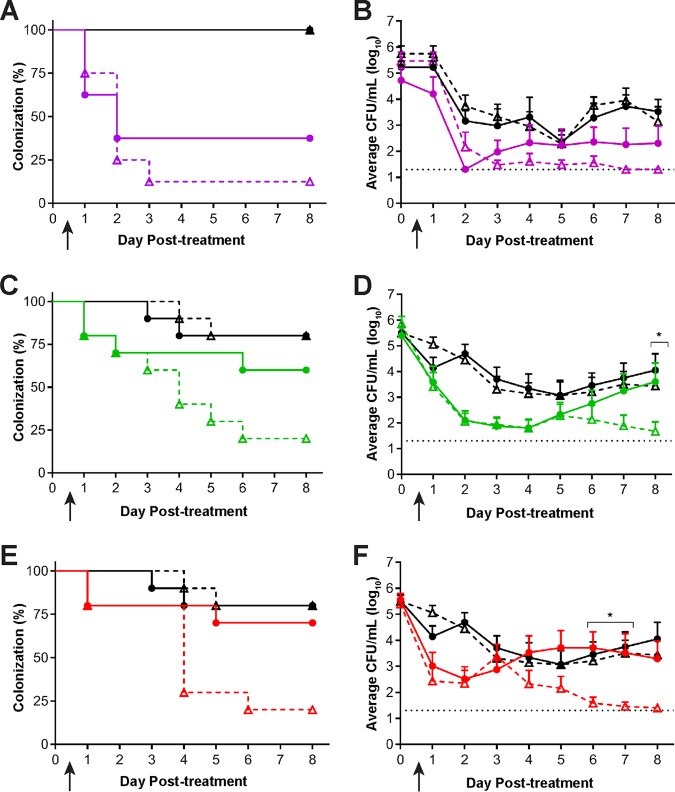
A dose response was observed following administration of PEN in mice infected with SC6 compared to the results for mice infected with SC7. Mice infected vaginally with SC7 (circles) or SC6 (triangles) were treated on day 0 (arrow) with PEN in 120 mg/kg TID (purple), 90 mg/kg TID (green), or 60 mg/kg TID (red) dosing regimens (*n* = 7 to 10 mice/group). PBS TID (black) was administered as a negative control. (A and B) Administration of 120 mg/kg PEN TID significantly reduced both SC6 and SC7 infection as shown by the percentage of mice infected (*P < *0.01) (A) and the average bacterial burden recovered (B) compared to the results for the PBS negative control (*P < *0.03). (C and E) Administration of PEN at 90 mg/kg TID (C) or 60 mg/kg TID (E) significantly reduced the percentage of SC6-infected mice compared to the results for the PBS negative control group (*P < *0.047), but the percentage of SC7-infected mice was comparable to the results for the negative control (*P > *0.3). (D and F) The average bacterial burden recovered for SC6-infected mice treated with PEN at either 90 mg/kg TID (D) or 60 mg/kg TID (F) was significantly reduced compared to the results for PBS alone (*P = *0.02) over the course of infection; however, the bacterial burden recovered from SC7-infected mice treated with either dose was comparable to the results for the negative control (*P > *0.2). *, *P < *0.03 for indicated days where SC6-infected mice that received PEN had a reduced bacterial burden compared to the results for PEN-treated SC7-infected mice.

Based on our results with CRO, we next sought to determine whether PEN could be used to effectively clear SC6 *in vivo*. As described above, under inducible conditions, SC6 is 8-fold more susceptible to PEN *in vitro* than SC7, which is greater than the 4-fold difference in CRO MICs against these strains. Although PEN follows the same PK parameter (*fT*_MIC_) as CRO and other β-lactam antibiotics (21), PK studies have not been conducted for PEN in the gonorrhea mouse model. We therefore utilized the reported lower plasma protein binding for PEN (8.5%) compared to that of CRO (60%) in mice, combined with the modeled PK data for CRO (19, 21), to predict the *fT*_MIC_ for PEN in the mouse model. The *fT*_MIC_s for a 120-mg/kg TID dose of PEN against SC6 and SC7 were similar (22.9 and 24.9 h, respectively); a 60-mg/kg TID dose was predicted to drop the *fT*_MIC_ to 18.1 h for SC7 versus 24.9 h for SC6. We next conducted a dose-response experiment in which groups of SC6- or SC7-infected BALB/c mice were treated with 60, 90, or 120 mg/kg PEN TID or with PBS. The 120-mg/kg TID dose cleared infection by either strain significantly compared to the results for PBS, with 63% of SC7-infected mice and 88% of SC6-infected mice cleared of infection by 72 h posttreatment (PEN versus PBS, *P* = 0.01 for SC7 and *P* = 0.001 for SC6) (Fig. 4A). Lower doses of PEN did not show efficacy against SC7 (PEN versus PBS, 90 mg/kg PEN TID, *P* = 0.3, and 60 mg/kg PEN TID, *P* = 0.6) (Fig. 4C and E). In contrast, both of these regimens were effective against strain SC6 (PEN versus PBS, 90 mg/kg or 60 mg/kg PEN TID, *P* ≤ 0.005) (Fig. 4C and E). Quantitative analysis of CFU recovered from infected mice showed that each of these PEN doses significantly reduced the CFU of SC6 compared to the results for the PBS negative control group (*P = *0.02), while the results for SC7 were comparable to the results for the negative control (Fig. 4B, D, and F).

With the emergence of gonococcal strains resistant to front-line antibiotics (AZM and CRO) used in dual therapy for gonorrhea in many countries and historical precedents for the ability of this sexually transmitted pathogen to develop resistance to every antibiotic brought into clinical practice, new treatment approaches must be considered. Herein, we demonstrate through *in vitro* antibiotic susceptibility testing and an experimental infection and treatment model that genetic dampening of the expression of the *mtrCDE* efflux pump operon in a β-lactam-resistant clinical isolate (H041) can render gonococci susceptible to PEN, based on the CLSI breakpoint (29), and decrease CRO resistance. This conclusion is consistent with earlier work (7) showing that loss of the MtrCDE efflux pump in H041 resulted in PEN susceptibility even though the genetic derivative contained and expressed other beta-lactam resistance determinants (e.g., mosaic *penA* and mutations in *porB* and *ponA*). An added benefit of the loss or reduced levels of MtrCDE would be increased susceptibility to host defense compounds, such as cationic antimicrobial peptides, bile salts, and progesterone (22–24). In this respect, genetic inactivation of the *mtrCDE* operon rendered gonococci unable to survive in the lower genital tract of experimentally infected female mice (25), which suggests that the MtrCDE efflux pump is a virulence factor that promotes gonococcal survival during infection. Thus, adjunctive therapies that target the MtrCDE efflux system could enhance clearance of gonococci by both antibiotics and host-derived antimicrobials.

This work was facilitated by our recent findings that PK modeling for CRO can predict the *in vivo* efficacy of this antibiotic against CRO-susceptible and -resistant gonococci (19). As an extension of the use of this model, we employed PEN to learn if it is theoretically possible to overcome resistance to this historically important antibiotic. We reasoned that infections caused by non-beta-lactamase-producing gonococci that harbor chromosomal resistance genes (e.g., mosaic *penA*) could be cleared if the expression of the *mtrCDE* efflux could be dampened and if the *fT*_>MIC_ could be sustained. Our findings support this concept and suggest that the developing EPIs that target the MtrCDE efflux pump and alternative dosing regimens would allow for a return of PEN to the clinic for treatment of gonorrhea. Additionally, through such efforts, the longevity of ESCs used in the clinic for this purpose could be extended.

## MATERIALS AND METHODS

### Bacterial strains and growth conditions.

The N. gonorrhoeae strains used for this study are listed in Table 1. Gonococcal strains were grown in GCB broth containing defined supplements I and II (26) or on GCB agar with supplements under 5% CO_2_ (vol/vol) at 37°C as described previously (26). When indicated in Results, isopropyl-β-d-thiogalactopyranoside (IPTG) was added to GCB broth or agar (final concentration of 1 mM).

### Cloning and expression of *mtrR* and strain construction.

The WT *mtrR* gene from strain FA19 was introduced into strain H041 using the pGCC4 vector as previously described (27). pGCC4, which contains an IPTG-regulated *lac* promoter (17), was digested with PacI and PmeI and purified by agarose gel electrophoresis. The *mtrR* coding sequence and 38 bp of upstream DNA were PCR amplified from chromosomal DNA prepared from strains FA19 or KH16 (FA19 *mtrR45*) using oligonucleotide primers 5′*mtrR* and 3′*mtrR* (Table S2 in the supplemental material) and ligated into PacI-/PmeI-digested pGCC4. The ligation reaction was used to transform Escherichia coli strain DH5α as described previously (27). Plasmid DNA was prepared from a representative transformant, and the correct *mtrR* coding sequence was confirmed by DNA sequencing using 5′*mtrR* and 3′*mtrR*. After verification of the *mtrR* coding sequence, the plasmid construct was used to transform N. gonorrhoeae strain H041 with selection for resistance to erythromycin (4 μg/ml). A representative transformant obtained using the WT *mtrR* coding sequence was retained and termed SC4. The presence of *mtrR* between *lctP* and *aspC* was determined by DNA sequencing of PCR products obtained using oligonucleotide primers LctP_F and 5′*mtrR* (Table S2). IPTG induction of *mtrR* expression was determined as described below.

10.1128/mBio.01576-19.3 TABLE S2 Oligonucleotides used in this study. Download Table S2, PDF file, 0.5 MB. Copyright © 2019 Chen et al. 2019 Chen et al. This content is distributed under the terms of the Creative Commons Attribution 4.0 International license.

The *mtrD* gene of strain SC4 was inactivated using genomic DNA from strain KH14 (FA19 *mtrD*::*kan* [20]) as described previously; gene inactivation was confirmed by PCR using oligonucleotide primers MTRD1 and KanC (Table S2). A resulting transformant was retained and termed SC5. To prepare strains SC6 and SC7, genomic DNA from FA19 *rpsL* was used to transform strains SC4 and H041, respectively, for resistance to streptomycin (100 μg/ml).

### Antimicrobial susceptibility testing.

The MICs of different CRO, PEN, and TX-100 concentrations were determined by spotting approximately 10^5^ CFU of N. gonorrhoeae suspensions onto GC agar containing antimicrobials with 2-fold differences in concentration; the MIC was defined as the lowest concentration at which bacterial growth was not observed after 48 h of incubation as described above. All antibiotics and chemicals were obtained from Sigma Chemical Co. (St. Louis, MO).

### Detection of MtrR and MtrE.

Strains FA19, H041, and SC4 were grown overnight on GC plates as described above. Bacterial growth was recovered using sterile cotton swabs and resuspended into GC broth to an optical density at 600 nm of 1.0. One-milliliter aliquots of each sample were pelleted and washed with PBS. The final pellet was resuspended in 200 μl of 2× Laemmli solubilizer (containing 8% β-mercaptoethanol) and boiled for 5 min (28). The solubilized samples were subjected to sodium dodecyl sulfate-polyacrylamide gel electrophoresis (10% [wt/vol] gels) as described previously (9) and then stained with Coomassie brilliant blue to ensure equal loading of protein samples (ca. 10 μg) or subjected to immunoblotting. MtrR and MtrE were detected using polyclonal rabbit antiserum (1/1,000 dilution) as described previously (9, 24).

### RNA isolation and qRT-PCR.

Total RNA was isolated from gonococcal cultures grown to mid-log phase using TRIzol (Invitrogen) as described previously (9, 27). Purified RNA was treated with the Turbo DNA-free kit (Ambion), and cDNA was generated using a QuantiTect reverse transcriptase kit (Qiagen). The levels of target genes were determined by quantitative PCR in a 25-μl SYBR green (Bio-Rad) reaction mixture using 2 μl of diluted cDNA as the template. The normalized expression of each target was calculated by the 2^−ΔΔ^*^CT^* threshold cycle (*C_T_*) method using 16S rRNA as a housekeeping reference gene. Mean fold change values were equivalent to the normalized expression ratio.

### *In vivo* efficacy testing.

For infection and treatment studies, bacteria were propagated on GC agar (BD Biosciences) supplemented with Kellogg’s supplement and 12 μM Fe(NO_3_)_3_ under 7% CO_2_ at 37°C (20). GC agar containing vancomycin, colistin, nystatin, trimethoprim, and streptomycin (GC-VCNTS agar) was used to isolate gonococci from mice as described previously (26). Female BALB/c mice (6 to 7 weeks old, NCI BALB/c strain; Charles River Laboratories) in the diestrus or anestrus stages of the estrous cycle were implanted with a 5-mg, 21-day slow-release 17β-estradiol pellet (Innovative Research of America) and treated with antibiotics to promote long-term gonococcal infection according to a standard infection protocol (20). Mice were inoculated vaginally with 20 μl of 10^4^ CFU H041 or SC6 (Str^r^) suspended in 20 μl of PBS on day −2. Vaginal swab samples were quantitatively cultured on day −1 and day 0 following bacterial inoculation; following culture on day 0, CRO (10 ml/kg intraperitoneally [i.p.]) and PEN (10 ml/kg i.p.) were administered to mice infected with H041 or SC6 as either two (BID; every 12 h) or three (TID; every 8 h) doses of antibiotic over a 24-h period. PBS was the negative control used for both H041 and SC6 infections and was administered by i.p. injection (10 ml/kg TID). Mice were weighed on day 0 prior to antibiotic administration, and preparations of CRO, PEN, or the PBS negative control were administered as 10-ml/kg doses of stocks of antibiotic prepared to achieve the desired test concentration. Ceftriaxone disodium salt hemi(heptahydrate) (CRO) and penicillin G sodium salt (PEN) were prepared in sterile endotoxin-free distilled water (dH_2_O) and delivered by i.p. injection. Vaginal swab samples were quantitatively cultured for N. gonorrhoeae for 8 consecutive days posttreatment as described previously (19). Mice were considered to have cleared the infection when vaginal cultures were negative (no CFU recovered) for three or more consecutive days. The limit of detection was 20 CFU/ml; this value was used in the data analysis for mice from which no gonococci were recovered. Differences in the duration of colonization were assessed using a Kaplan-Meier survivorship curve and log rank (Mantel-Cox) test. Differences in colonization load were assessed by a repeated-measures 2-way analysis of variance (ANOVA), using the Bonferroni test as a *post hoc* analysis for multiple pair-wise comparisons. All analyses were performed with GraphPad Prism version 7.05.

### Animal use assurances.

At the study endpoint, mice were euthanized using compressed CO_2_ gas in a CO_2_ gas chamber in the Laboratory Animal Medicine Facility. All animal experiments were conducted at the Uniformed Services University of the Health Sciences, a facility fully accredited by the Association for the Assessment and Accreditation of Laboratory Animal Care, under a protocol that was approved by the USUHS Institutional Animal Care and Use Committee.
