# Supplementary material for: Could Dampening Expression of the Neisseria gonorrhoeae mtrCDE-Encoded Efflux Pump Be a Strategy To Preserve Currently or Resurrect Formerly Used Antibiotics To Treat Gonorrhea?
Source: mBio. 2019 Aug 13;10(4):e01576-19. doi: 10.1128/mBio.01576-19 (PMC6692510; doi:10.1128/mBio.01576-19)
Supplement: FIG S1 [file mBio.01576-19-sf001.pdf]

Figure S1

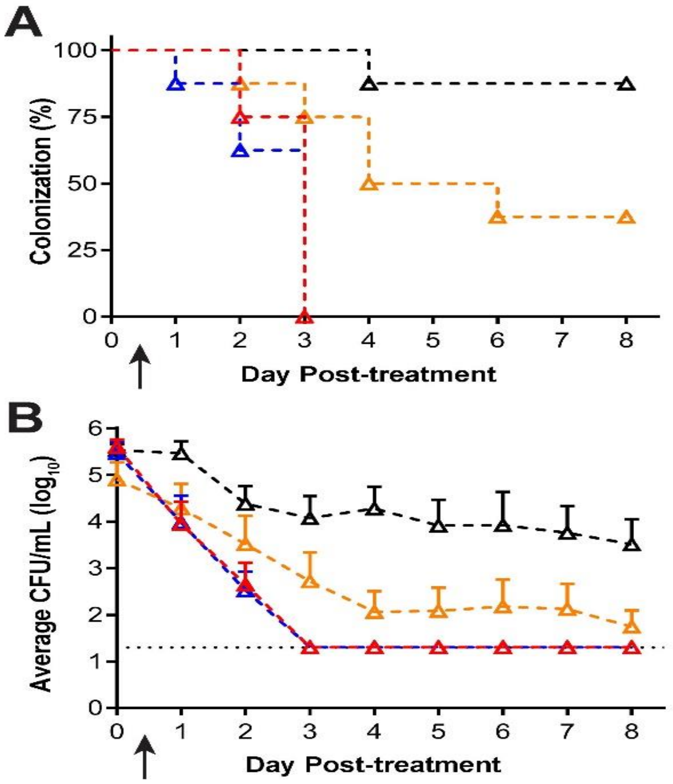

Figure S1. Dose response for in vivo clearance of S6. Mice infected vaginally with SC6 were treated on day 0 (arrow) with CRO as 60 mg/kg TID (red), 120 mg/kg BID (blue), or 30 mg/kg BID (orange) (n = 8 mice/group). PBS, TID (black) was administered as a negative control. Administration of all treatment regimens (A) significantly reduced both SC6 infection by (A) the percentage of mice infected ($p < 0.04$) and (B) the average bacterial burden recovered compared to the PBS negative control ($p < 0.004$), however, the 30 mg/kg BID treatment did not clear infection in all mice.
