# Supplementary material for: Could Dampening Expression of the Neisseria gonorrhoeae mtrCDE-Encoded Efflux Pump Be a Strategy To Preserve Currently or Resurrect Formerly Used Antibiotics To Treat Gonorrhea?
Source: mBio. 2019 Aug 13;10(4):e01576-19. doi: 10.1128/mBio.01576-19 (PMC6692510; doi:10.1128/mBio.01576-19)
Supplement: TABLE S1 [file mBio.01576-19-st001.pdf]

Table S1. Gene expression ratio of different genes under the induction of IPTG/without IPTG in SC4

Target genes	Expression change±SD (IPTG+/IPTG-)	Significance*
mtrR	86.34±11.22	<0.001
mtrC	0.3±0.06	<0.001
mtrE	0.32±0.12	<0.001
farR	0.53±0.05	<0.001
rpoH	0.46±0.08	<0.001
penA	1.09±0.17	0.376
porB	1.3±0.4	0.267
gyrA	0.99±0.25	0.898
norM	0.94±0.08	0.254

*P value was calculated by Student's t-test
