# Supplementary material for: Could Dampening Expression of the Neisseria gonorrhoeae mtrCDE-Encoded Efflux Pump Be a Strategy To Preserve Currently or Resurrect Formerly Used Antibiotics To Treat Gonorrhea?
Source: mBio. 2019 Aug 13;10(4):e01576-19. doi: 10.1128/mBio.01576-19 (PMC6692510; doi:10.1128/mBio.01576-19)
Supplement: TABLE S2 [file mBio.01576-19-st002.pdf]

TABLE S2. Oligonucleotides used in this study

Oligonucleotide	Sequence (5'→3')
5'mtrR	GGTTAATTAACCGCCCTCGTCAAACCGA
3'mtrR	GGGTTTAAACTTATTTCCGGCGCAGGCAG
MTRD1	CGGCATCTGAAGCCAAACCTGC
KanC	GTGGTATGACATTGCCTTCTGCG
LctP_F	CGGTCCGATTCAGCAGCAA
mtrR_qRT_F	CTTGTTTGACGCGTTGTTCCA
mtrR_qRT_R	GTGGATGTCGTTGCTTTGCA
mtrC_qRT_F	CGGATTTGGCGCGTTACAAA
mtrC_qRT_R	TAATGCGCGAACGGTTCAGA
mtrE_qRT_F	TGTCTGCCTGCACCATGATT
mtrE_qRT_R	AGTGCGATGTCGATCAGCTT
farR_qRT_F	AGCCCTGATGACCCAATTCAG
farR_qRT_R	TTTCAAGGCGGGTCAGGATAC
rpoH_qRT_F	AACGGCAGCCTCGAACAATA
rpoH_qRT_R	GGTGGGACAGGATGAGTTGTT
penA_qRT_F	AGGTTGCCGAAGAGGTCAAA
penA_qRT_R	TTCCTGACCTTTGCCGTCAA
porB_qRT_F	ACAATCCTTCGTCGGCTTGA
porB_qRT_R	TCCAGCACATTGCCGGTAAA
gyrA_qRT_F	AAACTGACTCCGCTGCAAGA
gyrA_qRT_R	TTCATGGCGTGCCTTCTTCA
norM_qRT_F	ATGCTGCTCGACCTCGACC
norM_qRT_R	CATCACGGTATCGACGAAACCGATGCCC
macA_qRT_F	AATTCGACCACGCAGACCAA
macA_qRT_R	CTGCGCGCTTTCCAAATCTT
16S_qRT_F	GTAGGGTGCGAGCGTTAATC
16S_qRT_R	CATCGGTATTCCTCCACATCTC

*Primers with ending letters qRT_F or qRT_R were used in qRT-PCR (see Table S1)
